# An enhanced version of FREM (Fracture Risk Evaluation Model) using national administrative health data: analysis protocol for development and validation of a multivariable prediction model

**DOI:** 10.1186/s41512-023-00158-w

**Published:** 2023-10-03

**Authors:** Simon Bang Kristensen, Anne Clausen, Michael Kriegbaum Skjødt, Jens Søndergaard, Bo Abrahamsen, Sören Möller, Katrine Hass Rubin

**Affiliations:** 1 Research Unit OPEN, Department of Clinical Research, University of Southern Denmark, Heden 16, Odense C, 5000 Denmark; 2 OPEN - Open Patient data Explorative Network, Odense University Hospital, Odense, Denmark; 3 Department of Public Health, Aarhus University, Aarhus, Denmark; 4 Department of Medicine, Holbæk Hospital, Holbæk, Denmark; 5 Research Unit of General Practice, Department of Public Health, University of Southern Denmark, Odense, Denmark

**Keywords:** Automated risk calculation, Machine learning, Prediction algorithm, Decision support tool, Osteoporotic fractures, Register data, Primary care, General practice, Decision aid, LASSO regularization, Gradient-boosted classification trees

## Abstract

**Background:**

Osteoporosis poses a growing healthcare challenge owing to its rising prevalence and a significant treatment gap, as patients are widely underdiagnosed and consequently undertreated, leaving them at high risk of osteoporotic fracture. Several tools aim to improve case-finding in osteoporosis. One such tool is the Fracture Risk Evaluation Model (FREM), which in contrast to other tools focuses on imminent fracture risk and holds potential for automation as it relies solely on data that is routinely collected via the Danish healthcare registers. The present article is an analysis protocol for a prediction model that is to be used as a modified version of FREM, with the intention of improving the identification of subjects at high imminent risk of fracture by including pharmacological exposures and using more advanced statistical methods compared to the original FREM. Its main purposes are to document and motivate various aspects and choices of data management and statistical analyses.

**Methods:**

The model will be developed by employing logistic regression with grouped LASSO regularization as the primary statistical approach and gradient-boosted classification trees as a secondary statistical modality. Hyperparameter choices as well as computational considerations on these two approaches are investigated by an unsupervised data review (i.e., blinded to the outcome), which also investigates and handles multicollinarity among the included exposures. Further, we present an unsupervised review of the data and testing of analysis code with respect to speed and robustness on a remote analysis environment. The data review and code tests are used to adjust the analysis plans in a blinded manner, so as not to increase the risk of overfitting in the proposed methods.

**Discussion:**

This protocol specifies the planned tool development to ensure transparency in the modeling approach, hence improving the validity of the enhanced tool to be developed. Through an unsupervised data review, it is further documented that the planned statistical approaches are feasible and compatible with the data employed.

## Background

Osteoporosis is a prevalent disease which represents a considerable and growing public health problem. The number of patients with osteoporosis in Europe is projected to rise from 27.5 million in 2010 to 33.9 million in 2025, corresponding to an increase of 23%, as a result of the increasing number of elderly in the population [1].

Furthermore, osteoporosis causes debilitating fragility fractures which are significant causes of mortality and morbidity for patients [2–4]. It is estimated that the annual number of osteoporotic fractures in Europe will rise from 4.28 million fractures in 2019 to 5.34 million fractures in 2034, corresponding to an expected increase of 24.8% [5]. Even though osteoporosis represents a significant healthcare burden on both patients and society, patients at risk of fracture do not receive appropriate treatment, resulting in a large treatment gap [6]. Results from the ICUROS study showed that among patients who sustained a low-energy hip fracture in 10 countries (Australia, Austria, Estonia, France, Italy, Lithuania, Mexico, Russia, Spain, and the UK), only 27% were prescribed fracture prevention treatment following the fracture, even though prior fracture is a known risk factor of subsequent fracture [7], showing that even secondary prevention is lacking [8]. Insufficient case-finding strategies cause osteoporosis to remain underdiagnosed and undertreated [1]. The treatment gap in osteoporosis therefore calls for improved detection of high-risk individuals.

Several fracture risk assessment tools have been developed to provide risk stratification of patients [9]. Such tools integrate known risk factors of osteoporosis and fracture into an estimate of fracture risk for patients. Some of the most commonly used tools include the fracture risk assessment tool (FRAX) [10] and the Garvan Institute of Medical Research—Bone Fracture Risk Calculator [11]. FRAX and Garvan use a set of cross-sectional clinical risk factors to estimate fracture risk, which typically requires manual data entry from physicians to retrieve fracture risk predictions. Furthermore, these tools predict long-term fracture risk, thus excluding patients with high imminent fracture risk.

However, new minimal physician–effort strategies are needed to systematically detect high-risk individuals. The Fracture Risk Evaluation Model (FREM) is a fracture risk assessment model that was developed to identify individuals at high imminent (1 year) risk of fracture, using hospital diagnoses from Danish registries and age as predictors. Predictors were chosen solely on the basis of their statistical predictive performance, without any assumptions on causality or biological relationship between predictor and outcome. Further, the model was developed for each sex, separately, to account for sex differences in fracture risk [12]. FREM exclusively utilizes routinely collected administrative health data from the Danish national registers, and the model therefore lends itself towards automatic risk predictions. FREM identified 38 and 43 hospital diagnoses in women and men, respectively, as risk factors for major osteoporotic fracture (MOF). Further, FREM identified 32 hospital diagnoses in both women and men as risk factors of hip fracture (HF). FREM showed good accuracy in prediction of MOF resulting in AUC = 0.750 (95% CI 0.741; 0.795) and AUC = 0.752 (95% CI 0.743; 0.761) for women and men, respectively. FREM showed even higher accuracy in prediction of HF resulting in AUC = 0.874 (95%CI 0.869; 0.879) and AUC = 0.851 (95% CI 0.841; 0.861) for women and men, respectively [12]. A validation study of FREM was recently performed in an updated extraction of Danish registry data which still proved good accuracy of FREM in prediction of MOF and HF [13]. Additionally, FREM has been externally validated in Canadian hospitalization and physician claims data proving significant fracture risk stratification [14]. FREM has the potential to be integrated into the primary health care system and can—without any manual data entry required by the general practitioners—provide a single easy-to-interpret estimate for each patients’ risk of MOF and HF. FREM has recently been suggested in international publications as a practicable future code of practice to identify patients at very high risk of osteoporotic fractures [15].

However, FREM holds the potential for further improvements in fracture risk prediction through the inclusion of data from additional health registries and the utilization of more advanced statistical methods including approaches from machine learning in model development. In particular, positive predictive values of the original FREM are fairly low [13], which is in accordance with an algorithm predicting a low prevalence outcome but also indicates a potential for reducing the number of false positives implied by FREM. Moreover, the original development utilized stepwise selection methods for identifying risk factors and employed data-splitting for assessing model performance, both techniques for which more modern equivalents have been developed [16].

Therefore, the aim of this article is to describe the development of an enhanced version of FREM and the evaluation of its performance in a Danish context.

The article follows the reporting guidelines set forth in the TRIPOD statement [17].

## Design and methods

### Data and data management

Data currently consists of the entire Danish population aged 45 or older on January 1, 2018, coupled with a 15-year look-back (2003–2017) in the Danish national registries, and these data are used in the analyses reported in the current article. We intend to update the data to include later years, before performing the analyses outlined in this protocol. Included individuals are observed for a period of 1 year from the index date, during which outcomes may occur. The 15-year look-back is used to collect information on personal characteristics and exposures, and we refer to these collectively as predictors. Other fracture prediction studies employ cohorts that are slightly younger (40 years and older [18–20]) or older [21–23]. Trémollieres and colleagues argue that a cutoff at 45 years allows for the inclusion of early menopausal women, who may be at increased risk of osteoporotic fracture [24]. The sample size is dictated by the study design and the data obtained from the Danish national registries. The original FREM study included a sample of 2,495,339 individuals [12].

Data sources largely coincide with those used for the original FREM tool [12], except that the present model also utilizes information on medicine redemptions and uses an updated data extraction from the registries. All data management and analyses are performed on a secure server at the Danish Health Data Authority.

### Data sources

Data is extracted from a collection of Danish health registries. The Danish health registries cover all citizens of Denmark and offer long-term data on the entire population and may generally be characterized as having a high degree of validity and completeness [25]. Data from the following three national registries are used in this study:

- The **Danish Civil Registration System** (**CRS**) includes all individuals residing in Denmark [26]. Upon birth or immigration, an individual is assigned a unique person identification number which may be used to identify the individual across registries [27]. The CRS is used to identify the study population and to determine age and sex.
- The **Danish National Patient Register** (**NPR**) is an administrative register containing all inpatient, outpatient, and emergency department contacts from Danish hospitals, since 1977 (outpatient and emergency departments since 1995) for both public and private hospitals (private hospitals since 2003) [28]. Records contain information on clinical characteristics along with procedures and treatment, in particular the diagnosis (-es) associated to the contact. The NPR is used to identify the outcomes of interest along with predictors using ICD-10 (International Classification of Diseases 10th Revision)-coding.
- The **Danish National Prescription Registry** (**DNPR**) contains individual-level data on all dispensed prescription pharmaceuticals sold in Danish community pharmacies [29]. Data comprises information on ATC (Anatomical Therapeutic Chemical Classification System)—codes, price reimbursement, etc., at the redemption level. The DNPR is used to define predictors.

### Outcome definitions

Two outcomes will be considered, a major osteoporotic fracture (MOF) or a hip fracture (HF). The primary outcome is MOF, defined as fracture of hip, clinical vertebral, wrist, or humerus (given as a primary or secondary ICD-10 code in the NPR: S120, S121, S122, S220, S221, S320, T08, S422, S423, S720, S721, S722, S525, or S526) [12] (Table 1). The secondary outcome is HF, which is given as a primary or secondary ICD-10 code in the NPR (S720, S721, or S722) [12] in combination with a surgical code (KNFB, or one of its sub-codes, or KNFJ4x-9x) [30]. Both definitions of MOF and hip fractures will be evaluated in a forthcoming medical record review and will potentially be updated following the results of this review.

**Table 1:** Outcome definition of MOF with ICD-10 coding

Fracture site	ICD-10	ICD-10 explanation
MOF		
Hip fracture	S720	Fracture of neck of femur
	S721	Pertrochanteric fracture
	S722	Subtrochanteric fracture
Clinical vertebral fracture	S120	Fracture of first cervical vertebra
	S121	Fracture of second cervical vertebra
	S122	Fracture of other specified cervical vertebra
	S220	Fracture of thoracic verbetra
	S221	Multiple fractures of the thoracic spine
	S320	Fracture of lumbar vertebra
	T08	Fracture of the spine, level unspecified
Wrist fracture	S525	Fracture of lower end of radius
	S526	Fracture of the lower end of both ulna and radius
Humerus fracture	S422	Fracture of the upper end of the humerus
	S423	Fracture of shaft of humerus

### Diagnoses as predictors

Information is collected on all non-administrative level 3 ICD-10 codes. We record both the occurrence and date of each ICD-10 code. If more than one instance of the same diagnosis occurs in the look-back period, the first record is used. Note that previous experiences of an osteoporotic fracture thus enter as predictors through the ICD10 codes and that those with previous fractures are not excluded. The diagnosis exposure is defined as the time from the first diagnosis to the end of the look-back period.

Rare diagnoses are excluded based on the unsupervised review as described below.

### Pharmacological exposures from redemptions

Redemptions (i.e., prescriptions filled) are perceived as relevant risk predictors in two ways. First, the redemption may act as a proxy for a diagnosis that is not recorded through the administrative health registers under consideration. This may occur since the NPR contains information on diagnoses from hospital contacts only and not from general practice consultations. Diagnosis registrations from general practice are difficult to retrieve for research purposes as they are not registered in a national database but in systems provided by privately owned software providers. However, data on redemptions of prescription drugs prescribed by general practitioners can be retrieved from the DNPR and redemptions may therefore act as proxies for diagnoses from general practice. Secondly, the redemption may signify exposure to a pharmaceutical with effects on fracture risk, which may influence the risk of outcome by e.g., affecting the individual’s bone density or by altering the risk of experiencing a serious fall. The second class of pharmaceuticals with effects on fracture risk are further subsets depending on their assumed type of effect. We record whether the ATC code was redeemed during the look-back period and further record a measure of the extent of exposure. The following pharmaceutical exposures are used:

1. Medications associated with risk of developing osteoporosis: Defined by the ATC codes in Table 2 (taken from [31]). Exposure is coded as time (in days) from the earliest redemption in the look-back period and defined as zero if no redemption occurred.
2. Fall-specific: Table 3 gives an overview of pharmaceuticals related to fall risk and the associated ATC codes. Their exposure is coded as 15 times 365.25 (i.e., the number of days in the lookback period) minus the number of days from the last redemption during the look-back period and is defined to be zero if no redemption occurred. Thus, a high exposure signifies a very recent redemption.
3. Diagnosis proxies: ATC codes to be used as proxies for diagnoses are all non-osteoporosis-specific codes included on the therapeutic level of the ATC hierarchy (level 3), meant to reflect the same therapeutic areas as the ICD-10 codes. For these ATC codes, we follow the principle described above for diagnoses and record the time of the first redemption. Exposure is the time from the first redemption to the index date (January 1, 2018). Note that a single redemption of an osteoporosis-specific drug may contribute both to the ATC-specific variable and to a diagnosis proxy. In the unsupervised review, we investigate any potentially ensuing problems of collinearity.

**Table 2:** Osteoporosis-specific medications

	ATC
Thiazolidinediones	A10BG
PPI	A02BC
Warfarin	B01AA03
Heparin	B01AB01
Glucocorticoids	H02AB
Cyclophosphamide	L01AA01
Methotrexate	L01BA01
Gonadotropin-releasing hormone (GNRH) agonists	L02AE
Aromatase inhibitors	L02BG
Calcineurin inhibitors	L04AD
Phenobarbital	N03AA02
Phenytoin	N03AB02
Carbamazepine	N03AF01
Valproate	N03AG01
SSRI	N06AB
Venlafaxine	N06AX16
Duloxetine	N06AX21

**Table 3:** Fall risk medications and associated ATC codes

Medications	ATC
Antiepileptics	N03 (all antiepileptics, including benzodiazepine derivate clonazepam)
Antidepressants	N06A
Antihypertensives	C02 (anti-adrenergic, alpha-blockers, vasodilators), C03 (thiazides, loop, potassium-sparring), C04 (peripheral vaso-dilators), C07 (beta-blockers), C08 (calcium-antagonists), C09 (RAAS-inhibitors)
Antipsychotics	N05A (all antipsychotics)
Antispasmodics	M03BX (baclofen, tizanidin)
Benzodiazepines and benzodiazepine-related drugs	N05BA (diazepam and related), N05CD, N05CF
Sedative hypnotics	N05C (all hypnotics)
Diuretics	C03, C07B, C07C, C09BA, C09DA
Sedative antihistamines	N07CA02 + N07CA52 (cinnarizin), R06AA02 (diphenhydramine), R06AA04 (clemastine), R06AE03 (cyclizin), R06AD02 (promethazine), R06AE05 (meclozin)
Vasodilators (used in cardiac disease), including alpha-blockers	C01DA, C01DX, C02
Overactive bladder and incontinence	G04BD12 (mirabegron), G04BD07 (tolterodin), G04BD10 (darifenacin), G04BD08 (solifenacin), G04BD11 (fesoterodin), G04BD09 (trospiumchlorid), G04CA
Anti-arrythmics	C01AA05, C01B
Anti-cholinergics	A03AB05, A03BA01, A03BA03, A03BB01, G04BD02, G04BD04, G04BD07, G04BD08, G04BD09, G04BD10, G04BD11, M03BC01, N04AA, N04AB, N04AC, N05AA01, N05AA03, N05AB03, N05AC02, N05AH03, N05AH04, N05BB, N06AA, N06AB05, R06AA

We retain all pharmaceutical exposures regardless of their prevalence of redemption.

### Loss to follow-up

We record loss to follow-up due to emigration prior to an outcome. This is expected to occur in only a very small fraction of the over-45-year-old population within a year, and we assume that any censoring of the outcome is the same as no fracture. Death before the outcome is treated as no fracture.

### Incomplete look-back

Not all included individuals have a full 15-year record in the registries, most commonly due to immigration. We record whether the look-back period is complete along with the number of years for which records could be obtained from the most recent immigration in the look-back period. An incomplete lookback period is likely to cause misclassification of risk factors as being absent, and we attempt to address this by including an incomplete lookback period as a predictor as detailed below.

### Prediction models

Analogous to the data management steps, analyses are performed on a secure server using R (version $\ge 4.1$) [32].

### Models

Let ${Y}_{i}^{\text{MOF}}$ and ${Y}_{i}^{\text{HF}}$ be indicators for subject $i$ experiencing a MOF or HF, respectively, in 2018 and let

$$X_i=\left(sex_i,age_i,ILB_i,{\mathrm{diag}}_i,{\mathrm{redempt}}_i\right)$$

be a row vector with predictors (age, sex, indicator for an incomplete look-back period (ILB), diagnoses, and redemptions in the look-back period) for subject $i$. Here, diagnoses and redemptions are themselves vectors. For a vector ${{v}}_{i}$ we denote by ${v}_{i,k}$ its $k$’th entry.

We consider two types of prediction models, the primary model being LASSO-regularized logistic regression and the secondary-boosted decision trees. The purpose of both is to predict the 1-year fracture risks.

$${\mathbb{P}}\left({Y}_{i}^{\text{MOF}}=1\hspace{0.25em}|\hspace{0.25em}{X}_{i}\right), \text{and }{\mathbb{P}}\left({Y}_{i}^{\text{HF}}=1\hspace{0.25em}|\hspace{0.25em}{X}_{i}\right),$$

using the predictors in ${X}_{i}$.

The main analysis may be characterized as an instance of refining a “strong learner” as represented by the full model. In the secondary analysis, we apply a different principle by combining many “weak” learners (decision trees) by using boosting, an approach that is often considered a type of machine learning. For both models, hyperparameters controlling complexity and flexibility (e.g. spline knots, number, and depth of boosted trees) were chosen with a view to computation time informed by the outcome-blinded code tests.

### Primary: logistic regression with grouped LASSO regularization

We describe our proposed model for MOFs, but the same approach is used for HFs. Note that in accordance with the original FREM model, sex is considered an effect modifier for all predictor effects.

The logistic regression model originally planned is,

$$\begin{array}{ll}\mathrm{logit}{\mathbb{P}}\left({Y}_{i}^{\text{MOF}}=1\hspace{0.25em}|\hspace{0.25em}{X}_{i}\right)& ={\alpha }_{se{x}_{i}}+{\beta }_{se{x}_{i}}IL{B}_{i}\\ & +{f}_{0,se{x}_{i}}\left(ag{e}_{i}-45\right)+\sum\limits_{k=1}^{{K}_{diagn}}{f}_{k,se{x}_{i}}\left(diag{n}_{i,k}\right)\\ & +\sum\limits_{k=1}^{{K}_{redempt}}{g}_{k,se{x}_{i}}\left(redemp{t}_{i,k}\right),\end{array}$$

where ${f}_{0}$, ${f}_{k}$, and ${g}_{k}$ are natural cubic splines (i.e., piecewise polynomial functions that are twice continuously differential, the polynomial being linear outside the outer knots and cubic inside). The spline function does not include an intercept and is determined by a number of parameters equal to the number of knots plus one. Due to the lack of intercept, whenever the exposure argument is zero, the spline is zero and consequently the intercept ${\alpha }_{sex}$ may be interpreted as the log-odds of MOF for an individual of sex $sex$, who is 45 years old, and has a complete look-back period in which no relevant diagnoses or redemptions were recorded.

It was realized a priori that most diagnoses and redemption exposures have high proportions of observations at zero (i.e., unexposed individuals). While this does not constitute a computational problem for the spline regression (as it would eg for fractional polynomials), it influences the choice of knots and perhaps the interpretation of the spline effect [33]. The choice of knots was informed from the unsupervised data review. Due to low prevalences of most diagnoses and redemptions, it was decided to use no inner spline knots (thus modeling the effects as linear due to the boundary constraint). We retain the spline on age using four knots.

The spline is estimated by expanding the function in its basis functions. We originally planned to apply grouped LASSO-regularization when estimating the logistic model, so that every basis function belonging to a specific spline was grouped together. This means that the effect of an exposure to a diagnosis or pharmaceutical is either selected or not in the sparse solution provided by the LASSO. However, numerical experimentation in the unsupervised data showed that the group-regularization was infeasible due to the size of the data set. Since there is only the age spline in the model, we instead apply ungrouped LASSO regularization. The amount of regularization is controlled by the parameter $\lambda$.

### Tuning the shrinkage parameter

The choice of the regularization parameter $\lambda$ is often referred to as “tuning” the model. We tune the LASSO model using the cross-entropy loss,

$$-y\mathrm{log}\left(\widehat{\pi }\right)-\left(1-y\right)\mathrm{log}\left(1-\widehat{\pi }\right),$$

for a class $y$ and predicted class probability $\hat{\pi}$. This is the negative log-likelihood of a binomial variable or, up to a constant, the deviance. Tuning is based on estimates of the loss function on a $\lambda$-grid. We use the default grid chosen by the software. To ameliorate overfitting, estimates are obtained from 10-fold cross-validation. The shrinkage parameter is then taken to be the $\lambda$ which minimizes the loss on the grid.

### Secondary: gradient-boosted classification trees

As above, we focus on the MOF outcome. The basic model is,

$${\mathbb{P}}\left({Y}_{i}^{\text{MOF}}=1\hspace{0.25em}|\hspace{0.25em}{X}_{i}\right)=h\left({X}_{i}\right),$$

where $h$ is a prediction rule that is to be determined by gradient-boosting classification trees [34]. A sequential method, boosting focuses on the part of the data that is not well-classified by the rule and adds a learner to better target this. Specifically, in gradient boosting, the new learner is chosen to predict the loss function’s gradient (corresponding intuitively to the part of the data that is not well predicted) and the rule is updated by weighting the new learner using a weight (the “step size”) estimated from the data. The learning rate may be dampened by introducing a multiplier for the step size.

As a loss function, we use the cross-entropy defined in [35]. Each learner is a classification tree of fixed depth (i.e., the number of terminal nodes). Since the primary analysis model includes no interactions, we take the tree depth to be 2, thus allowing each tree to contribute with up to two-factor interactions between predictors. The learning rate is chosen to be 0.1 with considerations of computational speed as investigated in the outcome-blinded code tests. Similarly, the number of trees is initially taken to be 1000, a choice which is updated using out-of-bag samples to estimate the loss function as a function of the number of trees. Aside from predictions from the model, we calculate the relative importance of the predictors in $X$ (as described in [34]).

### Evaluation of model performance

For both models, we consider measures of discriminative ability (how well does the algorithm separate those with fracture from those without fracture) as well as their ability to perform absolute risk prediction (how precisely does the model predict the fracture risks), i.e., the model’s calibration.

As a measure of discriminative ability, we use the area under the receiver operating characteristic curve (AUC), which may be interpreted as the probability that an individual with a fracture is assigned higher risk by the model than an individual with no fracture.

To assess calibration, we inspect calibration plots that arise by plotting observations against predicted probabilities and smoothing the relationship using a LOWESS smoother (e.g., [36]). Plots are constructed for the primary and secondary models and for both MOF and HF. As a measure of calibration, we further calculate the measure [16, 36],

$${E}_{\text{max}}\left(0,b\right)=\underset{0\le \widehat{\pi }\le b}{\mathrm{max}}|\widehat{\pi }-{\widehat{\pi }}_{c}|,$$

where $\widehat{\pi }$ and ${\widehat{\pi }}_{c}$ are the model-predicted risk probabilities and the corresponding value of the calibration curve, respectively. Thus, ${E}_{\text{max}}\left(0,b\right)$ is a measure of the largest discrepancy between the predicted and “true” risks in the range 0 to $b$. Varying $b$ shows calibration in different ranges of risks (where lower risks are presumed much more prevalent in the population). We plot the measure for increasing $b$ and report ${E}_{\text{max}}\left(0,1\right)$ as the primary statistic for calibration. As a supplementary measure of calibration, we use the integrated calibration index, which is the expected absolute discrepancy over the distribution of risk probabilities [36].

### Internal validation by bootstrapping

Non-parametric bootstrapping is used to perform interval validation [16, 37]. For both the AUC and ${E}_{\text{max}}\left(0,1\right)$ measure, the optimism due to overfitting is estimated: A bootstrap sample is formed from the data set by sampling with replacement, and the outlined analysis procedures are performed using the bootstrap sample as a training set and the original data as a test set. The performance measure in the training set minus that on the test set is the observed optimism, which is then estimated by averaging over the bootstrap samples. The apparent performance measure may then be corrected by subtracting the optimism.

Note that for the LASSO regression, the choice of lambda grid is also performed in each bootstrap sample.

### Unsupervised data review (i.e., blinded to the outcome)

The data management steps described above were performed on the raw data from the Danish Health Data Authority. Several considerations emerging from the review have already been reported above in their appropriate context.

Diagnosis exposures and diagnosis proxies from redemption data were formed. We excluded rare diagnoses and proxy diagnoses that had a prevalence below 0.1%. Generally, predictor prevalences were low, and consequently, it was decided to omit inner knots from splines (leading to a linear effect due to the boundary condition for the natural spline) to avoid overfitting the exposure effects. As described above, we modeled the age effect using 4 knots.

Investigations of collinearity revealed 11 predictors (6 medications and 5 diagnoses), which were highly collinear with other predictors and hence were removed from the selection procedure.

Following the blinded review of predictors, 785 potential predictors remained (including age and sex).

**Fig. 1:**
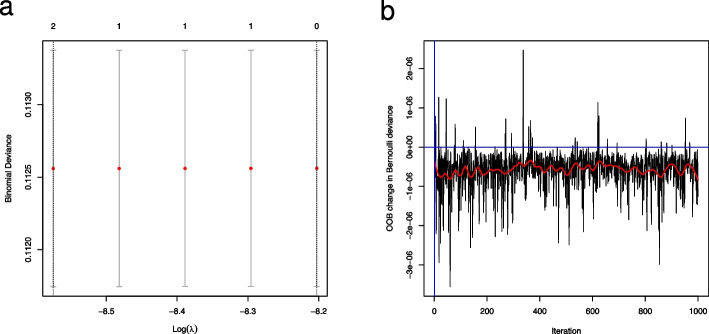
**a** Binomial deviance from the primary analysis of simulated outcome data. Binomial deviance as a function of the regularization parameter (on a logarithmic scale) in the LASSO regression as estimated by cross-validation. The figure contains point estimates as well as error bars signifying the standard error of the estimate. The numbers in the top of the figure denote the degrees of freedom in the model. Vertical dashed lines denote the smallest regularization estimate (leftmost line) and the smallest regularization plus one standard error. **b** Change in binomial deviance from secondary analysis of simulated outcome data. Out-of-bag (OOB) estimates of the change in binomial deviance as a function of the number of trees in the boosting procedure of the secondary analysis. The relationship is smoothed in the red curve, while vertical and horizontal blue lines demarcate the origin

To guide the choice of hyperparameters, the primary and secondary analyses described above were implemented, and the code was tested in an approach blinded to the outcome by simulating an outcome independently from all predictors with an overall prevalence of 1%. Figure 1 includes diagnostic plots for the primary and secondary analyses. Although based upon a single simulation, the results are promising as they show that there is no tendency of the methods to overfit the data: Both methods correctly identify no relevant predictors as they should since the outcomes have been simulated independently from risk factors.

The code was implemented in R version 4.1 using the glmnet ([38], version 4.1–4) package to fit the primary analysis and the gbm package ([34], version 2.1.8.1) for the secondary analysis. The mock analysis differed from those described above in that it was implemented on the entire data set, not stratified by sex.

## Discussion

In this protocol paper, we described the planned enhancement of the FREM tool by including additional data sources and applying a more advanced statistical methodology. Furthermore, the exposures and statistical methodology were investigated in an unsupervised data review.

The main strength of the planned study is building on top of an existing tool, which has already been validated in multiple settings [13, 14], hence ensuring that the resulting enhanced version of FREM will potentially improve on top of an already applicable tool. The enhanced FREM has the potential to be integrated into the primary health care system as a decision support tool to optimize the identification of individuals at high risk of MOF. Moreover, the use of administrative registry data including a complete population ensures a limited risk of selection bias in the tool development.

The main limitations of the planned study are the validity of exposures and outcomes obtained from administrative registries. While some exposures have been shown to be valid in the literature [28], this has not been investigated for all included exposures. The validity of outcomes will be investigated in a forthcoming medical record review, which will be concluded before the planned study is performed. Furthermore, the statistical methods planned and applied in the unsupervised data review make some assumptions on association structure, and misspecification of these models could influence the validity of the results. The planned approach of using two separate statistical approaches (LASSO and gradient-boosted classification trees) will ensure that model misspecification will be detectable as a discrepancy between those two strategies. Following the development of an updated version of FREM, external validations will be performed in future studies, both in different populations outside of Denmark, as well as temporal validations to investigate a possible drift in the model performance. These external validations are not part of the current protocol.

Results of the planned study will be reported in one or multiple scientific papers in international peer-reviewed journals, and deviations from this protocol, if any occur, will be pointed out in detail and substantiated in these publications.

In this paper, we have described the forthcoming development of an updated version of the FREM tool including additional data sources and more advanced statistical methods. We find that our approach will take into account 785 possible predictors after selecting against collinearity and sparsity. Moreover, our unblinded review has identified appropriate choices for the hyperparameters to be used in the development of an enhanced version of FREM.

## Data Availability

Data cannot be published due to existing personal data regulations. Data are stored on a secure server at the Danish Health Data Authority.

## Abbreviations

FREM: Fracture Risk Evaluation Model
FRAX: Fracture risk assessment tool
GIH-BFRC: Garvan Institute of Health Bone Fracture Risk Calculator
HF: Hip fracture
MOF: Major osteoporotic fracture
AUC: Area under the receiver operating characteristics curve
CI: Confidence interval
CRS: Danish Civil Registration System
NPR: Danish National Patient Register
DNPR: Danish National Prescription Registry
ATC: Anatomical Therapeutic Chemical Classification System
ILB: Incomplete look-back period
LASSO: Least absolute shrinkage and selection operator

## References

[1] Hernlund E, Svedbom A, Ivergård M, Compston J, Cooper C, Stenmark J (2013). Osteoporosis in the European Union: medical management, epidemiology and economic burden. A report prepared in collaboration with the International Osteoporosis Foundation (IOF) and the European Federation of Pharmaceutical Industry Associations (EFPIA). Arch Osteoporos.

[2] Johnell O, Kanis JA, Odén A, Sernbo I, Redlund-Johnell I, Petterson C (2004). Mortality after osteoporotic fractures. Osteoporos Int.

[3] Osnes EK, Lofthus CM, Meyer HE, Falch JA, Nordsletten L, Cappelen I (2004). Consequences of hip fracture on activities of daily life and residential needs. Osteoporos Int.

[4] Marques A, Lourenço Ó, da Silva JA (2015). The burden of osteoporotic hip fractures in Portugal: costs, health related quality of life and mortality. Osteoporos Int.

[5] International Osteoporosis Foundation. Key statistics for Europe. 2021. Available from: https://www.osteoporosis.foundation/facts-statistics/key-statistic-for-europe. 11.10.2022.

[6] Kanis JA, Svedbom A, Harvey N, McCloskey EV (2014). The osteoporosis treatment gap. J Bone Miner Res.

[7] Wintzell V, Ivergård M, Tankó LB, Barghout V, Svedbom A, Alekna V (2013). The resource use related to hip fractures based on data from ICUROS. Value in Health.

[8] Skjødt MK, Ernst MT, Khalid S, Libanati C, Cooper C, Delmestri A (2021). The treatment gap after major osteoporotic fractures in Denmark 2005–2014: a combined analysis including both prescription-based and hospital-administered anti-osteoporosis medications. Osteoporos Int.

[9] El-Hajj Fuleihan G, Chakhtoura M, Cauley JA, Chamoun N (2017). Worldwide Fracture Prediction. J Clin Densitom.

[10] University of Sheffield. FRAX - fracture risk assessment tool. Available from: https://www.sheffield.ac.uk/FRAX/. 11.10.2022.

[11] Garvan Institute. Bone fracture risk calculator. Available from: https://www.garvan.org.au/bone-fracture-risk. 11.10.2022.

[12] Rubin KH, Möller S, Holmberg T, Bliddal M, Søndergaard J, Abrahamsen B (2018). A new Fracture Risk Assessment Tool (FREM) based on public health registries. J Bone Miner Res.

[13] Skjødt MK, Möller S, Hyldig N, Clausen A, Bliddal M, Søndergaard J (2021). Validation of the Fracture Risk Evaluation Model (FREM) in predicting major osteoporotic fractures and hip fractures using administrative health data. Bone.

[14] Möller S, Skjødt MK, Yan L, Abrahamsen B, Lix LM, McCloskey EV (2022). Prediction of imminent fracture risk in Canadian women and men aged 45 years or older: external validation of the Fracture Risk Evaluation Model (FREM). Osteoporos Int.

[15] Curtis EM, Reginster JY, Al-Daghri N, Biver E, Brandi ML, Cavalier E (2022). Management of patients at very high risk of osteoporotic fractures through sequential treatments. Aging Clin Exp Res.

[16] Harrell FE (2001). Regression modeling strategies: with applications to linear models, logistic regression, and survival analysis.

[17] Collins GS, Reitsma JB, Altman DG, Moons KG (2015). Transparent reporting of a multivariable prediction model for Individual Prognosis or Diagnosis (TRIPOD): the TRIPOD statement. J Clin Epidemiol.

[18] Tamaki J, Iki M, Kadowaki E, Sato Y, Kajita E, Kagamimori S (2011). Fracture risk prediction using FRAX®: a 10-year follow-up survey of the Japanese Population-Based Osteoporosis (JPOS) Cohort Study. Osteoporos Int.

[19] Azagra R, Roca G, Encabo G, Aguyé A, Zwart M, Güell S (2012). FRAX® tool, the WHO algorithm to predict osteoporotic fractures: the first analysis of its discriminative and predictive ability in the Spanish FRIDEX cohort. BMC Musculoskelet Disord.

[20] Leslie WD, Majumdar SR, Morin SN, Lix LM, Schousboe JT, Ensrud KE (2018). Performance of FRAX in clinical practice according to sex and osteoporosis definitions: the Manitoba BMD registry. Osteoporos Int.

[21] Holloway-Kew KL, Zhang Y, Betson AG, Anderson KB, Hans D, Hyde NK (2019). How well do the FRAX (Australia) and Garvan calculators predict incident fractures? Data from the Geelong Osteoporosis Study. Osteoporos Int.

[22] Fraser LA, Langsetmo L, Berger C, Ioannidis G, Goltzman D, Adachi JD (2011). Fracture prediction and calibration of a Canadian FRAX® tool: a population-based report from CaMos. Osteoporos Int.

[23] Yun H, Delzell E, Ensrud KE, Kilgore ML, Becker D, Morrisey MA (2010). Predicting hip and major osteoporotic fractures using administrative data. Arch Intern Med.

[24] Trémollieres FA, Pouillès JM, Drewniak N, Laparra J, Ribot CA, Dargent-Molina P (2010). Fracture risk prediction using BMD and clinical risk factors in early postmenopausal women: sensitivity of the WHO FRAX tool. J Bone Miner Res.

[25] Schmidt M, Schmidt SAJ, Adelborg K, Sundbøll J, Laugesen K, Ehrenstein V (2019). The Danish health care system and epidemiological research: from health care contacts to database records. Clin Epidemiol.

[26] Pedersen CB (2011). The Danish Civil Registration System. Scand J Public Health.

[27] Schmidt M, Pedersen L, Sørensen HT (2014). The Danish Civil Registration System as a tool in epidemiology. Eur J Epidemiol.

[28] Schmidt M, Schmidt SA, Sandegaard JL, Ehrenstein V, Pedersen L, Sørensen HT (2015). The Danish National Patient Registry: a review of content, data quality, and research potential. Clin Epidemiol.

[29] Pottegård A, Schmidt SAJ, Wallach-Kildemoes H, Sørensen HT, Hallas J, Schmidt M (2017). Data resource profile: the Danish National Prescription Registry. Int J Epidemiol.

[30] Hjelholt TJ, Edwards NM, Vesterager JD, Kristensen PK, Pedersen AB (2020). The positive predictive value of hip fracture diagnoses and surgical procedure codes in the Danish Multidisciplinary Hip Fracture Registry and the Danish National Patient Registry. Clin Epidemiol.

[31] Skjødt MK, Ostadahmadli Y, Abrahamsen B (2019). Long term time trends in use of medications associated with risk of developing osteoporosis: nationwide data for Denmark from 1999 to 2016. Bone.

[32] R Core Team (2021). R: a language and environment for statistical computing.

[33] Sauerbrei W, Perperoglou A, Schmid M, Abrahamowicz M, Becher H, Binder H (2020). State of the art in selection of variables and functional forms in multivariable analysis-outstanding issues. Diagn Progn Res.

[34] Greenwell B, Boehmke B, Cunningham J, GBM developers (2020). Gbm: generalized boosted regression models.

[35] Hastie T, Tibshirani R, Friedman J. The elements of statistical learning: data mining, inference and prediction. 2nd ed. New York: Springer; 2009.

[36] Austin PC, Steyerberg EW (2019). The Integrated Calibration Index (ICI) and related metrics for quantifying the calibration of logistic regression models. Stat Med.

[37] Efron B (1983). Estimating the error rate of a prediction rule: improvement on cross-validation. J Am Stat Assoc.

[38] Friedman J, Hastie T, Tibshirani R (2010). Regularization paths for generalized linear models via coordinate descent. J Stat Softw.

